# Novel Swine Influenza Virus Reassortants in Pigs, China

**DOI:** 10.3201/eid1607.091881

**Published:** 2010-07

**Authors:** Yuhai Bi, Guanghua Fu, Jing Chen, Jinshan Peng, Yipeng Sun, Jingjing Wang, Juan Pu, Yi Zhang, Huijie Gao, Guangpeng Ma, Fulin Tian, Ian H. Brown, Jinhua Liu

**Affiliations:** Author affiliations: China Agricultural University, Beijing, People’s Republic of China (Y. Bi, G. Fu, J. Chen, J. Peng, Y. Sun, J. Wang, J. Pu, Y. Zhang, H. Gao, J. Liu);; Shandong Animal Disease Control Center, Jinan, People’s Republic of China (J. Chen, F. Tian, J. Liu);; Veterinary Laboratories Agency–Weybridge, Addlestone, Surrey, UK (I.H. Brown);; China Rural Technology Development Center, Beijing (G. Ma);; Chongwen Animal Health Inspection Institute, Beijing (J. Peng)

**Keywords:** Influenza, viruses, H1N1, H1N2, H3N2, reassortant, swine, dispatch

## Abstract

During swine influenza virus surveillance in pigs in China during 2006–2009, we isolated subtypes H1N1, H1N2, and H3N2 and found novel reassortment between contemporary swine and avian panzootic viruses. These reassortment events raise concern about generation of novel viruses in pigs, which could have pandemic potential.

Genetic characterization of pandemic (H1N1) 2009 virus has indicated that it may have derived from swine (*1*,*2*). However, because of the lack of systematic swine influenza surveillance, the generation pathway of the novel virus is uncertain. Therefore, we attempted to obtain more information about swine influenza viruses isolated from pigs.

## The Study

During December 2006–February 2009 in the People’s Republic of China, 3,546 samples from 3 main swine industry provinces—Fujian (765 samples), Guangdong (1,276 samples), and Shandong (1,505 samples)—were collected for influenza surveillance. Nasal and tracheal swab samples were collected from apparently healthy domestic pigs at abattoirs. Virus isolation and identification were performed as described (*3*). Of 29 strains of influenza A virus obtained, 19 were subtype H1N1, 1 subtype H1N2, and 9 subtype H3N2. Subtype H1N2 was isolated from diseased pigs in Guangdong Province in 2006; the others were isolated from healthy pigs. Isolation rates for subtypes H1N1 and H3N2 were 0.54% and 0.25%, respectively, indicating that subtype H1N1 viruses were predominant in the sampled pig population.

To determine genetic and antigenic characteristics, we conducted phylogenetic and antigenic analysis of all isolates. Cross–hemagglutination-inhibition showed that the subtype H3N2 viruses could be divided into 2 distinct antigenic groups (Table A1). Viruses of subtype H1 (swine/Shandong/101/2008, swine/Shandong/327/2008, and swine/Shandong/275/2009) reacted well with antiserum to the European avian-like swine virus, swine/FJ/204/2007, but not with antiserum to classical swine (H1N1) virus, swine/Guangdong/1/2005. The other 6 subtype H1N1 isolates reacted strongly with antiserum to swine/Guangdong/1/2005 (Table A1; Table A2), indicating that the antigenicity of the subtype H1N1 viruses could also be divided into 2 distinct antigenic groups. Subtype H1N2 virus (swine/Guangdong/1222/2006) had low reactivity with swine/Guangdong/1/2005 and swine/Fujian/204/2007 (Table A2), indicating that the antigenicity of subtype H1N2 isolate differed from that of classical and European avian-like swine viruses.

Phylogenetic analysis showed that the H3 hemagglutinin (HA) tree separated into avian and human lineages (Technical Appendix, panel A), implying that at least 2 independent H3 sublineages of virus prevail in pigs in China. Neuraminidase (NA) genes of the 9 subtype H3N2 and 1 subtype H1N2 isolates were located in distinct lineages (Technical Appendix, panel B). A cluster was formed by 4 strains of H3N2—swine/Fujian/43/2007, swine/Guangdong/811/2006, swine/Shandong/106/2007, and swine/Shandong/133/2007—and the cluster grouped with Eurasian avian (H9N2) viruses. Four H3N2 strains—swine/Guangdong/211/2006, swine/Guangdong/423/2006, swine/Guangdong/223/2006, and swine/Guangdong/968/2006—were located in the intermediate human sublineage represented by A/Beijing/39/75 (H3N2). One subtype H3N2 isolate, swine/Guangdong/7/2006, grouped closely with A/Moscow/10/99 (H3N2), and the subtype H1N2 isolate swine/Guangdong/1222/2006 shared close similarities with North American swine triple reassortant viruses (Technical Appendix, panel B). These findings showed that viruses of avian, intermediate human, and recent human N2 sublineages were prevalent in pigs in China.

Phylogenic analysis of subtype H1 HA showed that the 9 subtype H1N1 isolates were located in either the classical or European avian-like swine lineages (Technical Appendix, panel C). Swine/Guangdong/1222/2006, together with subtype H1N2 isolates from Hong Kong and subtype H1N2 strains from Guangxi, have a sister-like relationship with those of pandemic (H1N1) 2009 virus (Technical Appendix, panel C). Consistent with characteristics of HA genes, NA genes of the 6 influenza (H1N1) strains isolated belong to classical swine lineages (Technical Appendix, panel D). The other 3 isolates—swine/Shandong/101/2008, swine/Shandong/275/2008, and swine/Shandong/327/2008—together with pandemic (H1N1) 2009 virus, were located in the European avian-like swine group.

The polymerase acidic protein (PA) gene of swine/Guangdong/7/2006 (H3N2) was closely related to that of duck/Guangdong/12/2000 (H5N1) (Technical Appendix, panel G), and other internal genes of swine/Guangdong/7/2006 were located in the recent human subtype H3N2 lineages (Technical Appendix, panels E, F, and H–J). The matrix (M) gene of the 3 isolates—swine/Guangdong/211/2006 (H3N2), swine/Guangdong/223/2006 (H3N2), and swine/Guangdong/423/2006 (H3N2)—grouped in classical swine lineage (Technical Appendix, panel I), and other internal genes were located in intermediate human subtype H3N2 lineage (Technical Appendix, panels E–H and J). Except for the fact that the PA gene of swine/Guangdong/968/2006 and NA gene of swine/Guangdong/811/2006 are of the Eurasian H9N2 avian virus lineage (Technical Appendix, panels B and G), the other internal genes are located in the same lineages with the 3 viruses swine/Guangdong/211/2006, swine/Guangdong/223/2006, and swine/Guangdong/423/2006 (Technical Appendix, panels E–J). The polymerase basic protein 1 (PB1), PA, NP, and nonstructural (NS) genes of swine/Shandong/106/2007 (H3N2) and swine/Shandong/133/2007 (H3N2) belong to the Eurasian avian lineage grouping with the H9N2 viruses (Technical Appendix, panels F–H and J). The PB2 and M genes of the 2 isolates group in human subtype H1N1 lineage (Technical Appendix, panels E and I). The PA and M genes of swine/Fujian/43/2007 (H3N2) belong to recent human-like H3N2 virus lineages (Technical Appendix, panels G and I); the NS gene originates from European avian-like virus (Technical Appendix, panel J), and the PB2, PB1, and NP genes were located in the Eurasian avian lineages with subtype H9N2 viruses (Technical Appendix, panels E, F, and H). Except for the M gene, all other internal genes of swine/Guangdong/1222/2006 have a sister-like relationship with those of pandemic (H1N1) 2009 virus (Technical Appendix, panels E–J). The PB1 gene of swine/Shandong/275/2008 was an exception, grouping with Eurasian avian subtype H9N2 virus (Technical Appendix, panel F). All 6 internal genes of the 3 Shandong isolates were located in the European avian-like lineages (Technical Appendix, panel E–J). All 8 genes of the 6 subtype H1N1 Guangdong isolates formed 1 cluster and belonged to classical swine lineages (Technical Appendix, panels C–J), indicating that none of these viruses were recent reassortants (Table).

## Conclusions

Influenza A subtypes H1N1, H1N2, and H3N2 viruses co-circulate in China. Genetic analysis showed that the single subtype H1N2 virus and all subtype H3N2 viruses examined were either double- or triple-reassortant viruses, which have been rarely documented in China. Finding a gene fragment ostensibly of highly pathogenic avian influenza (H5N1) virus in a subtype H3N2 virus implies that subtype H5N1 viruses may be able to contribute genes to virus pathogenic processes in pigs. Moreover, European avian-like swine (H1N1) virus undergoes reassortment with avian (H9N2) viruses.

Some researchers have hypothesized that pigs may serve as hosts for genetic reassortment between human and avian influenza viruses (*4*). Our results show that subtypes H3N2 and H1N2 and 1 European avian-like swine (H1N1) virus were all derived from relatively recent reassortment events. The gene fragments of the subtype H3N2 viruses comprised those of human subtype H3N2 (A/Victoria/75-like and A/Moscow/99-like) and the strains H1N1 classical swine, Eurasian H5N1, and H9N2 avian. Infection of pigs with avian H5N1 and H9N2 viruses in China has been reported, and swine H1 and H3 viruses appear widely established in the pig population in China and elsewhere in Southeast Asia (*5*–*9*). These findings raise more questions about the generation of novel viruses, which may have zoonotic potential, in pigs.

Pandemic (H1N1) 2009 virus probably resulted from reassortment of recent North American influenza subtypes H3N2 and/or H1N2 swine viruses with Eurasian avian-like swine viruses (*2*). The current situation, therefore, presents continued risk for further reassortment of swine influenza virus in pig populations and continued spread of pandemic (H1N1) 2009 virus to pigs worldwide. Systematic influenza virus surveillance in pigs is needed in China.

## Supplementary Material

Technical Appendix Unrooted neighbor-joining phylogenetic trees of swine influenza A virus subtypes H3N2, H1N2, and H1N1..

## Appendix

**Table A1:** Antigenic characterization of influenza (H3N2) viruses isolated from pigs*

Virus	Hyperimmune rabbit serum, titer				
	A/JXdh/312/2006	Sw/GD/7/2006	Sw/GD/211/2006	Sw/GD/968/2006	Dk/BJ/40/2004
A/JXdh/312/06 (H3N2)	**640**	160	<10	<10	<10
Sw/GD/7/06 (H3N2)	160	**1,280**	<10	<10	<10
Sw/SD/133/07 (H3N2)	160	640	<10	<10	<10
Sw/SD/106/07 (H3N2)	160	640	<10	<10	<10
Sw/FJ/43/07 (H3N2)	160	640	<10	<10	<10
Sw/GD/211/06 (H3N2)	<10	<10	**640**	640	40
Sw/GD/968/06 (H3N2)	<10	<10	640	**640**	20
Sw/GD/223/06 (H3N2)	<10	<10	320	640	40
Sw/GD/423/06 (H3N2)	<10	<10	320	320	<10
Sw/GD/811/06 (H3N2)	<10	<10	320	320	<10
Dk/BJ/40/04 (H3N8)	<10	<10	<10	<10	**1,280**

*JXdh, Jiangxidonghu; sw, swine; GD, Guangdong; dk, duck; BJ, Beijing; SD, Shandong; FJ, Fujian. **Boldface** indicates hemagglutination-inhibition titers with homologous strains. Titers expressed as the reciprocal of the dilution end point given complete inhibition of hemagglutination.

**Table A2:** Antigenic characterization of influenza subtype H1 viruses isolated from pigs*

Virus	Hyperimmune rabbit serum, titer				
	SW/FJ/204/2007	SW/GD/1/2005	Dk/HB/843/2005	Sw/GD/1222/2006	A/GDlh/219/2006
Sw/FJ/204/07 (H1N1)	**1,280**	<10	640	80	≤10
Sw/SD/101/08 (H1N1)	1,280	<10	640	80	≤10
Sw/SD/327/08 (H1N1)	1,280	<10	640	80	≤10
Sw/SD/275/09 (H1N1)	1,280	<10	640	80	≤10
Sw/GD/1/05 (H1N1)	320	**1,280**	320	640	40
Sw/GD/33/06 (H1N1)	640	640	320	320	80
Sw/GD/109/06 (H1N1)	640	1,280	640	320	80
Sw/GD/322/06 (H1N1)	320	1,280	320	320	80
Sw/GD/328/06 (H1N1)	640	640	320	320	80
Sw/GD/611/06 (H1N1)	640	1,280	640	320	80
Sw/GD/628/06 (H1N1)	320	1,280	320	320	80
Sw/GD/1222/06 (H1N2)	320	320	640	**1,280**	<10
Dk/HB/843/05 (H1N2)	<10	<10	**560**	<	<10

*Sw, swine; FJ, Fujian; GD, Guangdong; dk, duck; HB, Hebei; A, avian; GDlh, Guangdongluohu; SD, Shandong. Boldface indicates hemagglutination-inhibition titers with homologous strains. Titers expressed as the reciprocal of the dilution end point given complete inhibition of hemagglutination.

**Table:** Genetic origin of swine influenza viruses*

Isolate	Gene							
	PB2	PB1	PA	HA	NP	NA	M	NS
Swine/GD/7/2006 (H3N2)	R-H	R-H	A-H5	R-H	R-H	R-H	R-H	R-H
Swine/SD/106/2007 (H3N2)	H-H1	A-H9	A-H9	R-H	A-H9	A-H9	H-H1	A-H9
Swine/SD/133/2007 (H3N2)	H-H1	A-H9	A-H9	R-H	A-H9	A-H9	H-H1	A-H9
Swine/FJ/43/2007 (H3N2)	A-H9	A-H9	R-H	R-H	A-H9	A-H9	R-H	S-A
Swine/GD/211/2006 (H3N2)	H-75	H-75	H-75	H-75	H-75	H-75	C	H-75
Swine/GD/223/2006 (H3N2)	H-75	H-75	H-75	H-75	H-75	H-75	C	H-75
Swine/GD/423/2006 (H3N2)	H-75	H-75	H-75	H-75	H-75	H-75	C	H-75
Swine/GD/811/2006 (H3N2)	H-75	H-75	H-75	H-75	H-75	A-H9	C	H-75
Swine/GD/968/2006 (H3N2)	H-75	H-75	A-H9	H-75	H-75	H-75	C	H-75
Swine/GD/1222/2006 (H1N2)	A-A	R-H	A-A	C	C	R-H	C	C
Swine/GD/611/2006 (H1N1)	C	C	C	C	C	C	C	C
Swine/GD/322/2006 (H1N1)	C	C	C	C	C	C	C	C
Swine/GD/33/2006 (H1N1)	C	C	C	C	C	C	C	C
Swine/GD/446/2006 (H1N1)	C	C	C	C	C	C	C	C
Swine/GD/109/2006 (H1N1)	C	C	C	C	C	C	C	C
Swine/GD/628/2006 (H1N1)	C	C	C	C	C	C	C	C
Swine/SD/327/2009 (H1N1)	S-A	S-A	S-A	S-A	S-A	S-A	S-A	S-A
Swine/SD/101/2008 (H1N1)	S-A	S-A	S-A	S-A	S-A	S-A	S-A	S-A
Swine/SD/275/2009 (H1N1)	S-A	A-H9	S-A	S-A	S-A	S-A	S-A	S-A

*PB, polymerase basic protein; PA, polymerase acidic protein; HA, hemagglutinin; NP, nucleocapsid protein; NA, neuraminidase; M, matrix; NS, nonstructural; GD, Guangdong; R-H, recent human-like (H3N2); A-H5, Eurasian avian (H5N1); SD, Shandong; H-H1, human-like (H1N1); A-H9, Eurasian avian (H9N2); FJ, Fujian; S-A, European avian-like swine; H-75, intermediate human-like (H3N2) (A/Victoria/75-like); C, classical swine; A-A, American avian.
