# Supplementary material for: Novel Swine Influenza Virus Reassortants in Pigs, China
Source: Emerg Infect Dis. 2010 Jul;16(7):1162–4. doi: 10.3201/eid1607.091881 (PMC3321907; doi:10.3201/eid1607.091881)
Supplement: Technical Appendix — Unrooted neighbor-joining phylogenetic trees of swine influenza A virus subtypes H3N2, H1N2, and H1N1.. [file 09-1881-Techapp.pdf]

DOI: 10.3201/eid1607.091881

Novel Swine Influenza Virus Reassortants in Pigs, China

Technical Appendix

The unrooted neighbor-joining phylogenetic trees on the following pages show swine influenza A virus subtypes H3N2, H1N2, and H1N1. Trees were generated by the Maximum Composite Likelihood model in software MEGA 4.1 (www.megasoftware.net). The reliability of the tree is assessed by bootstrap analysis with 1,000 replications; only bootstrap values ≥90% are shown. Horizontal distances are proportional to genetic distance. Blue, subtype H3N2 viruses isolated in this study; purple, H1N2 isolates; green, H1N1 isolates; red, human influenza A pandemic (H1N1) 2009 viruses. Underlining indicates viruses that were used to prepare polyclonal antiserum.

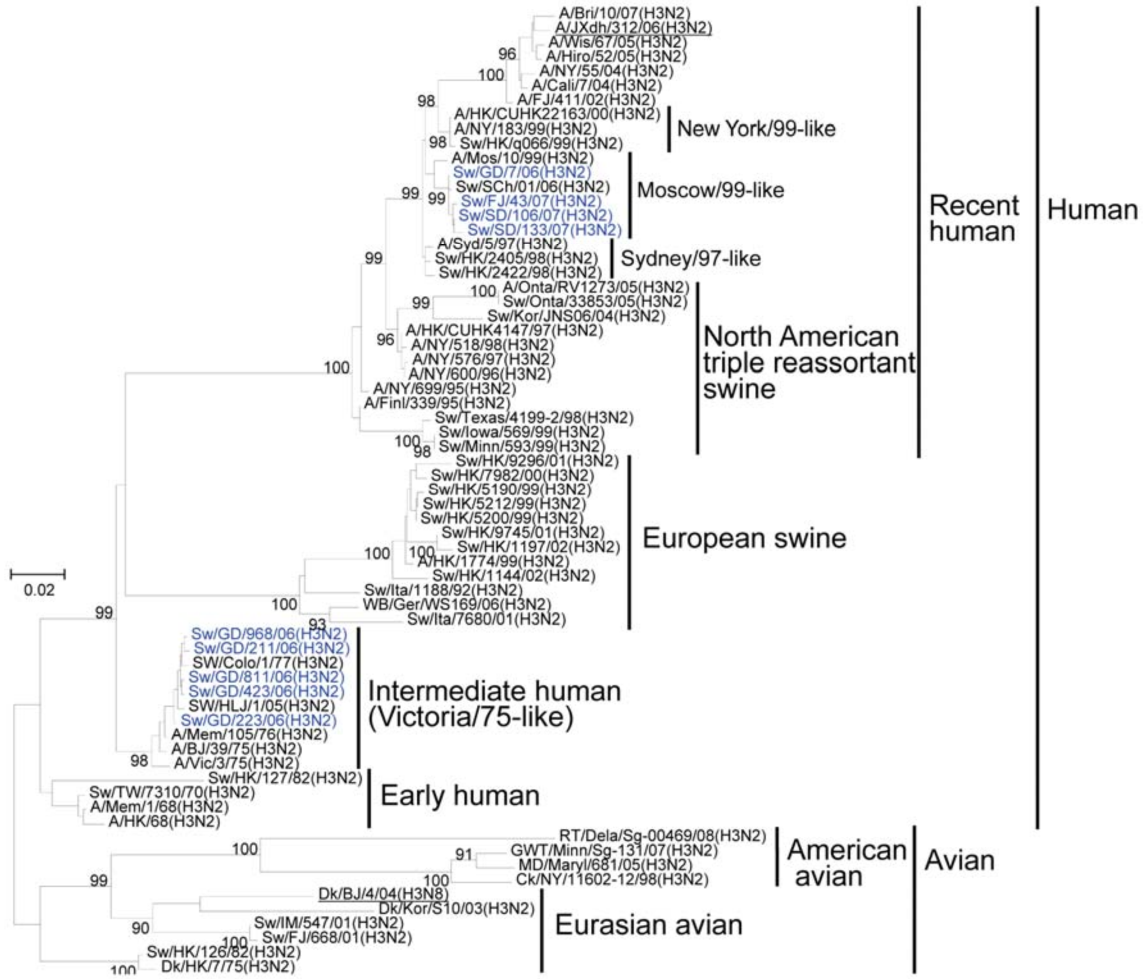

Hemagglutinin 3, nt 98–1064.

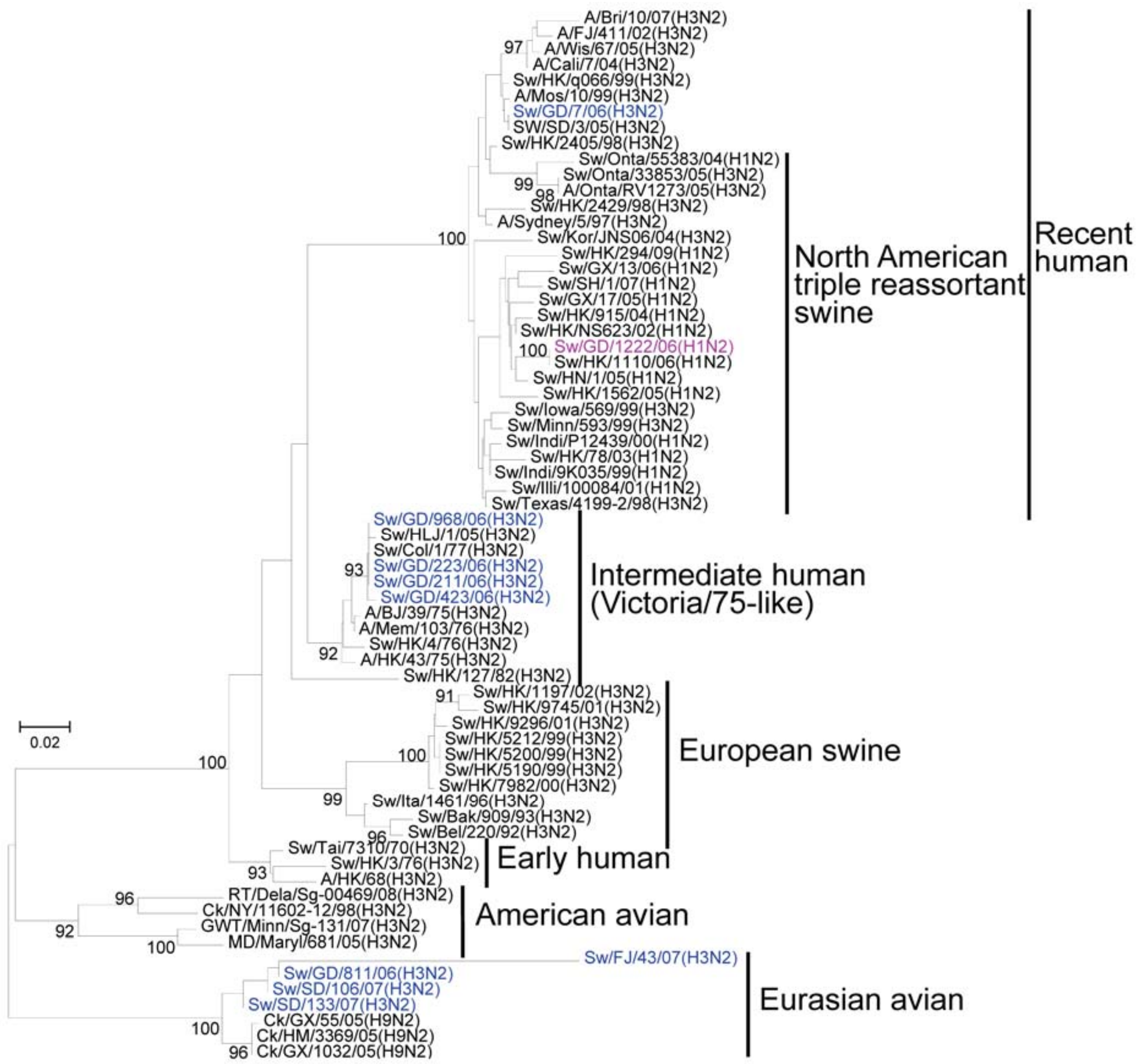

Neuraminidase 2, nt 28–1293.

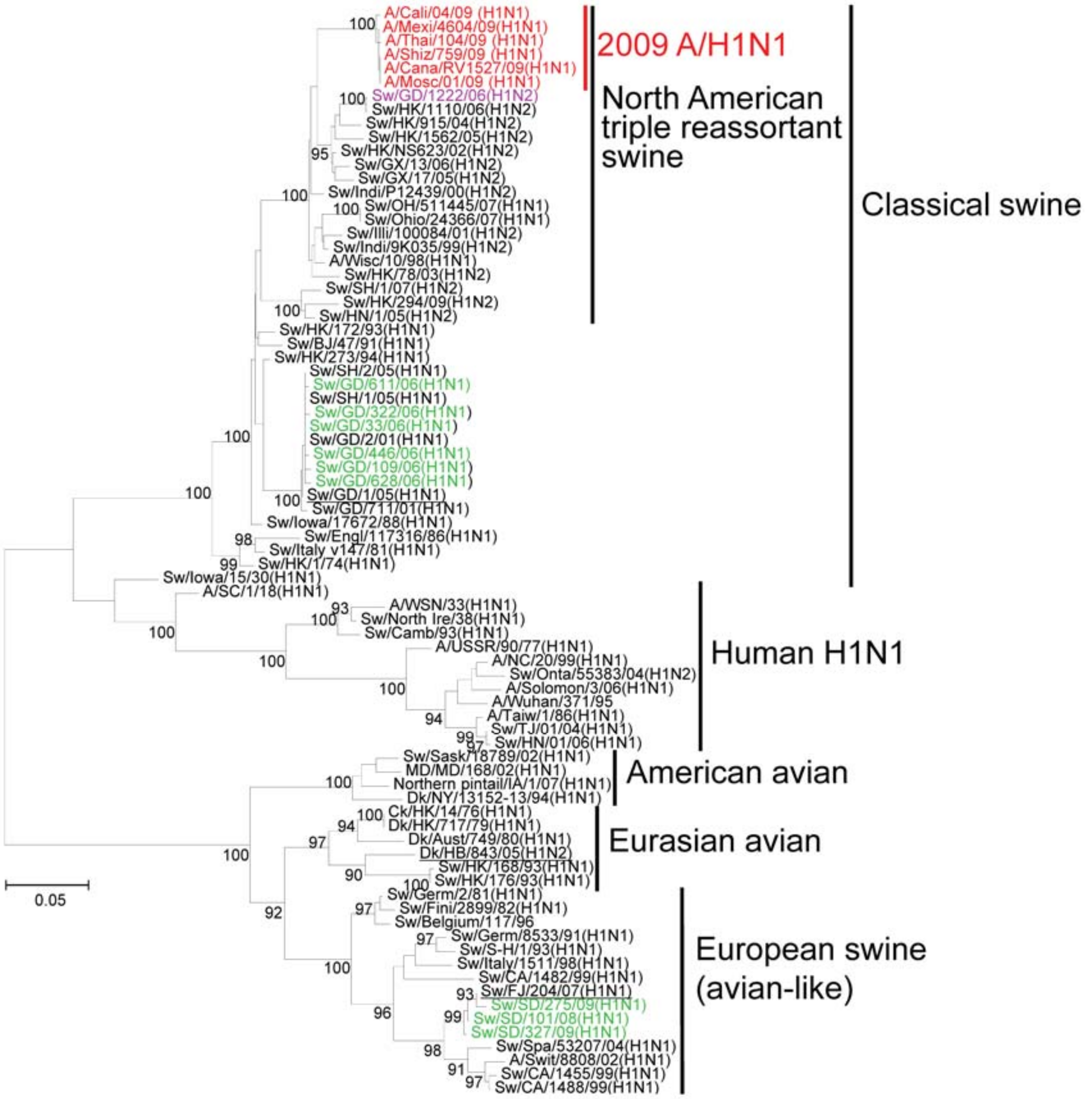

Hemagglutinin 1, nt 84–1052.

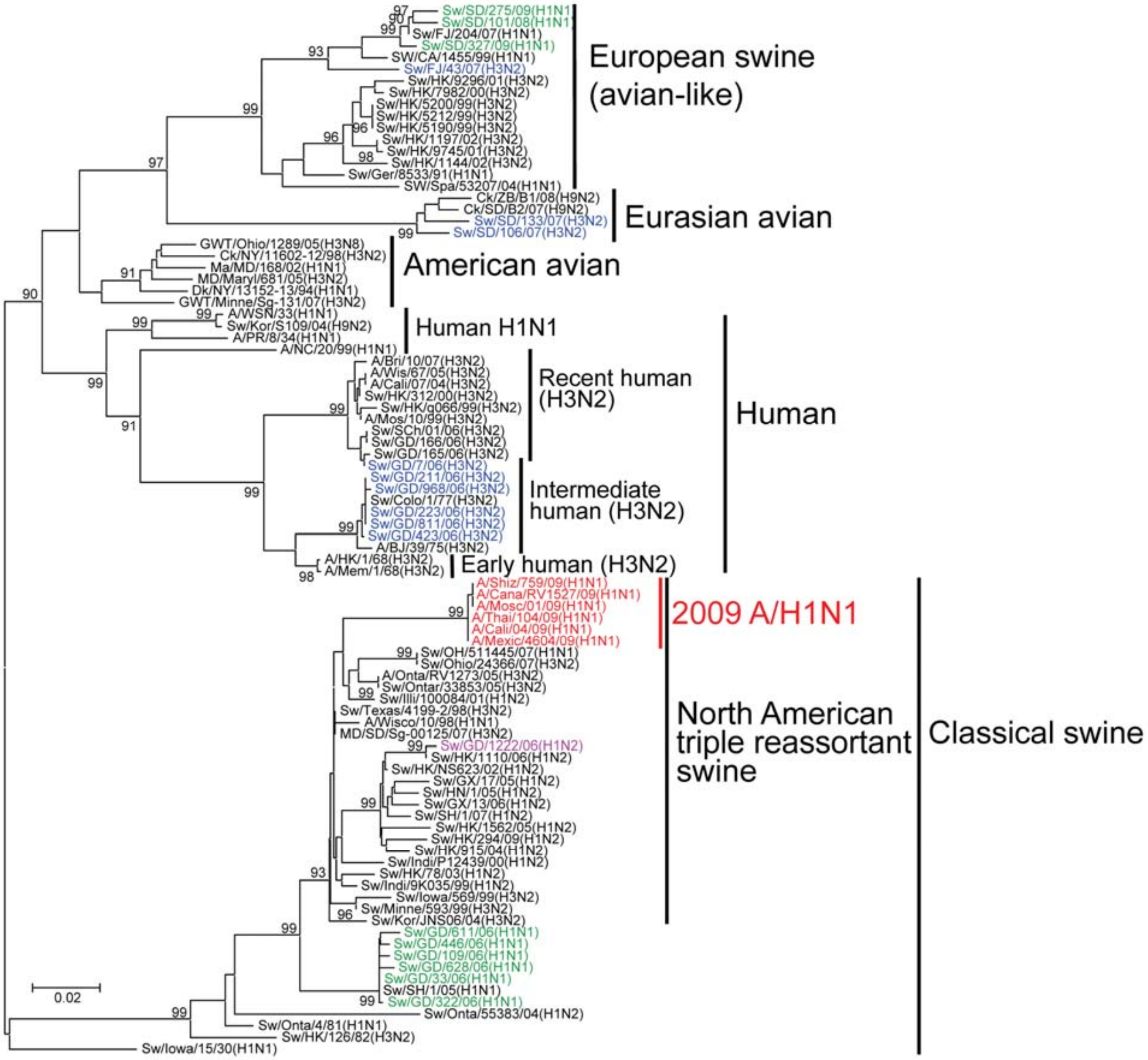

Neuraminidase 1, nt 21–1429.

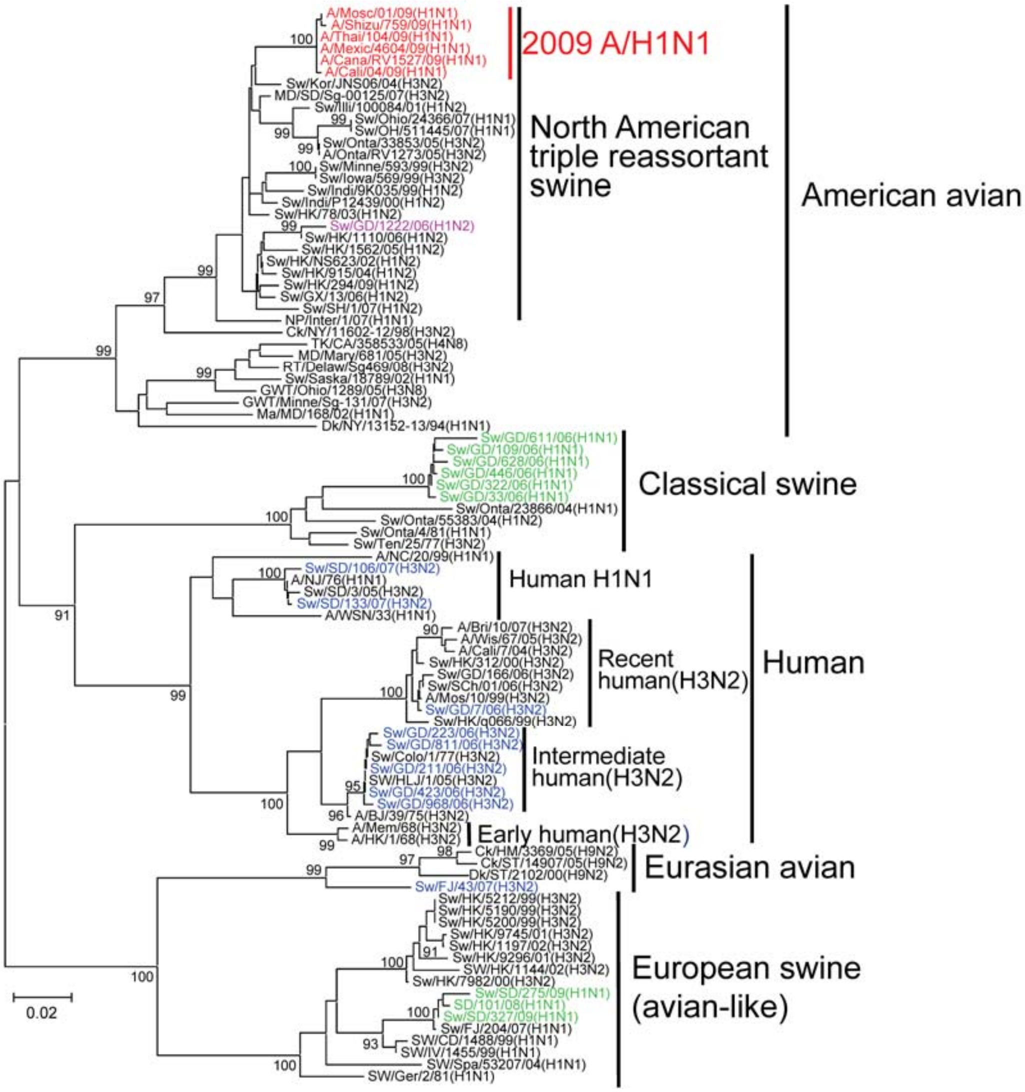

Polymerase basic protein 2, nt 1105–2273.

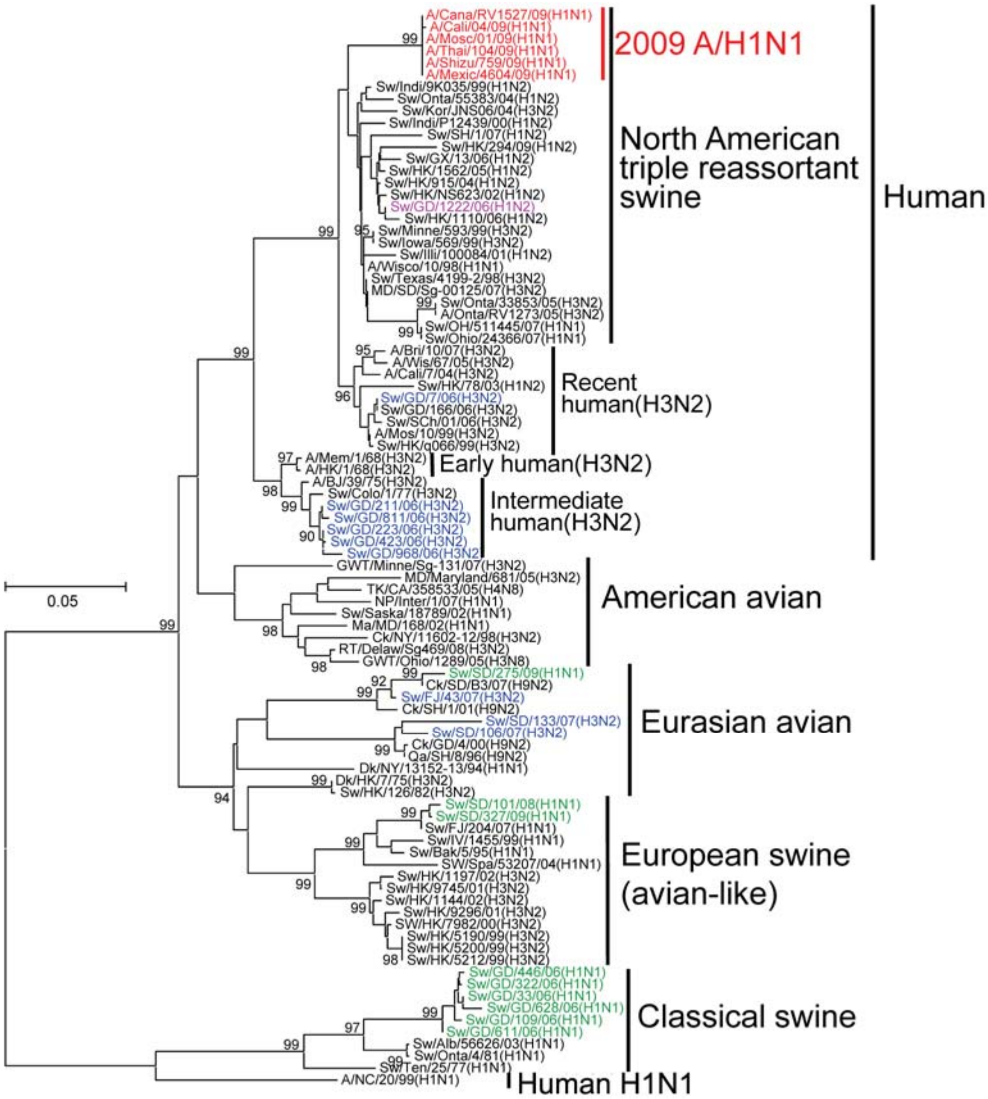

Polymerase basic protein 1, nt 52–1813.

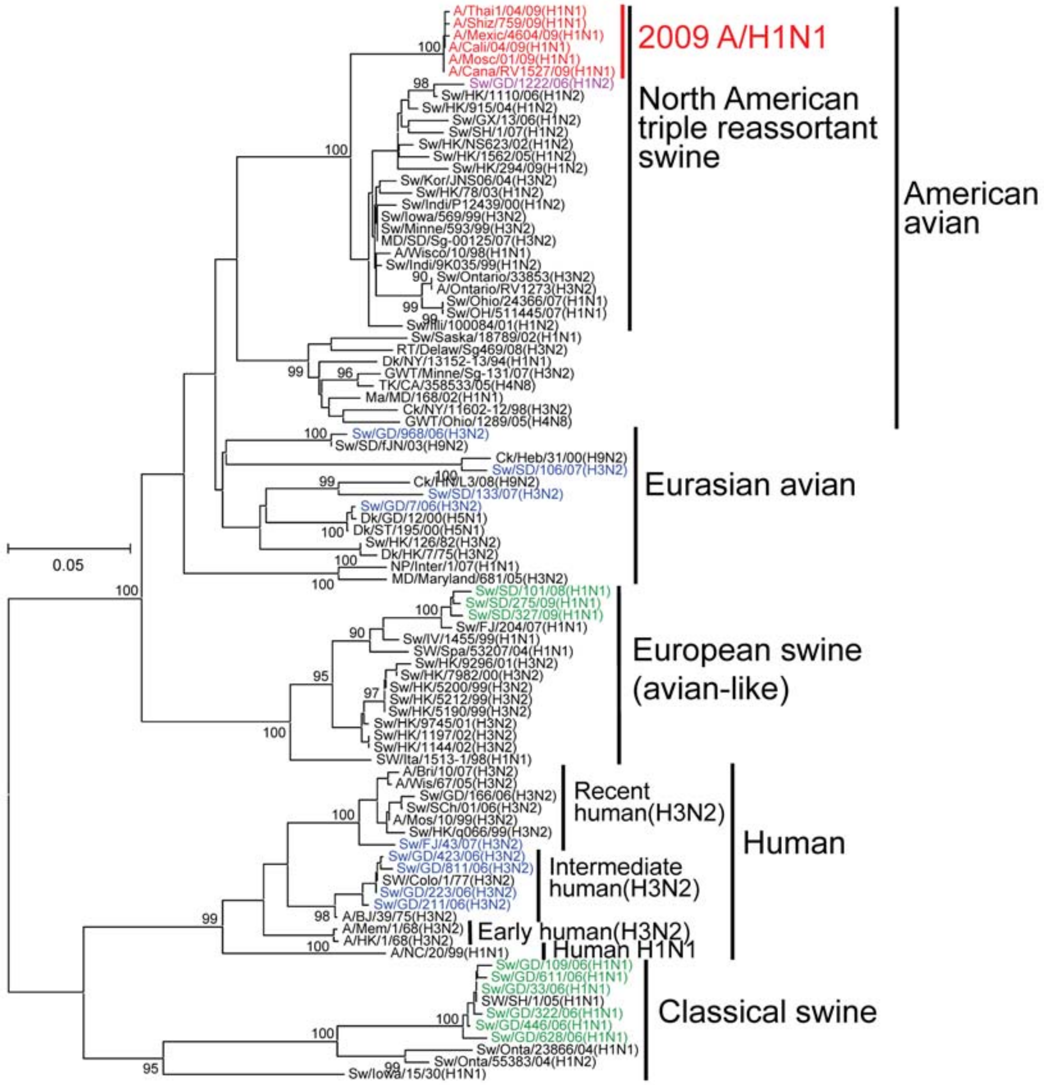

Polymerase acidic protein, nt 40–1251.

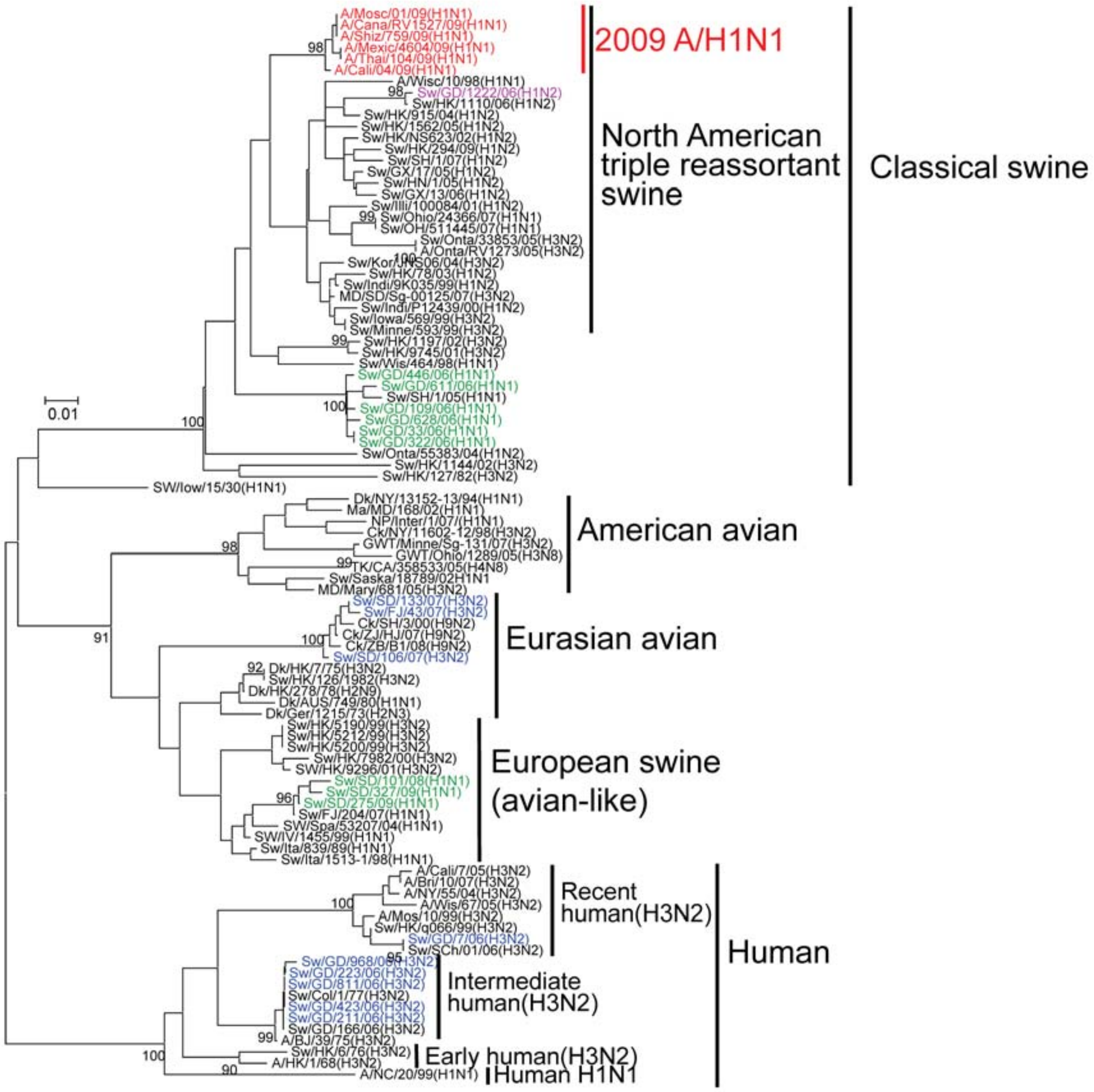

Nucleocapsid protein, nt 46–1520.

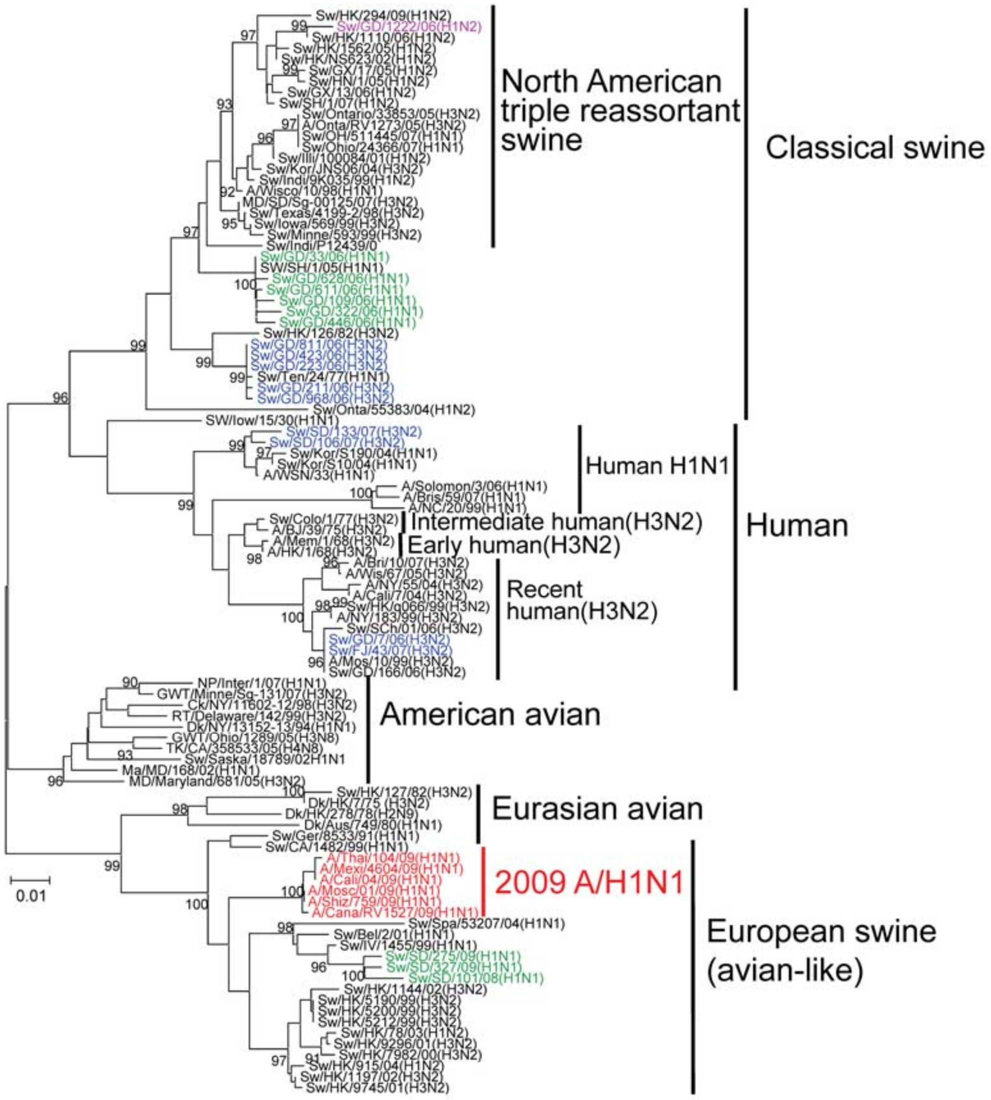

Matrix protein, nt 35–998.

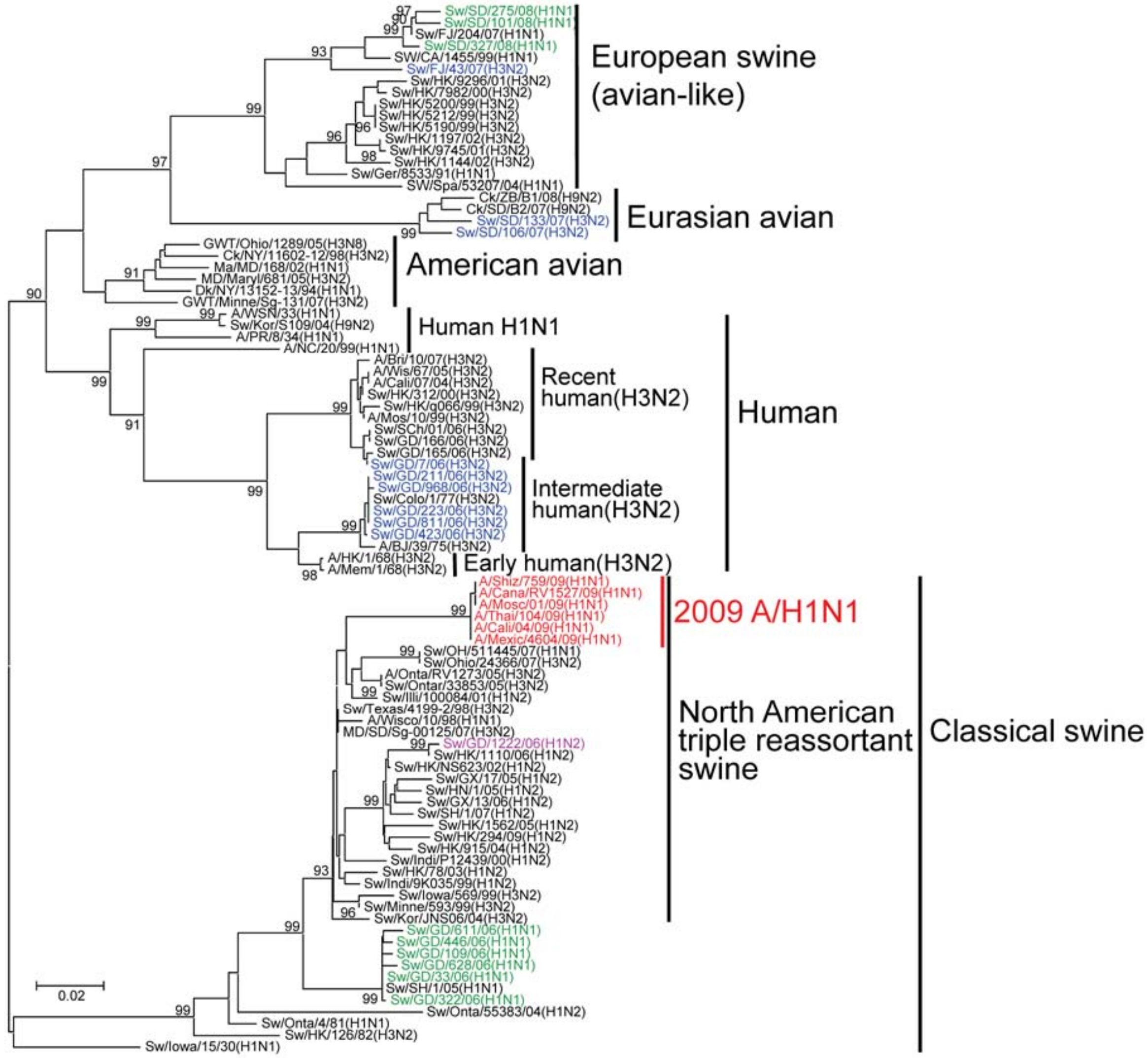

Nonstructural gene, nt 27–864.
